# Exosomes Derived From M2 Macrophages Facilitate Osteogenesis and Reduce Adipogenesis of BMSCs

**DOI:** 10.3389/fendo.2021.680328

**Published:** 2021-07-06

**Authors:** Ziyi Li, Yafei Wang, Shilun Li, Yukun Li

**Affiliations:** ^1^ Department of Endocrinology, The Third Hospital of Hebei Medical University, Shijiazhuang, China; ^2^ Department of Joint Surgery, The Third Hospital of Hebei Medical University, Shijiazhuang, China

**Keywords:** macrophage, bone marrow mesenchymal stem cell, exosomes, osteogenesis, adipogenesis

## Abstract

Bone regeneration is a complex process that requires the coordination of osteogenesis and osteoclastogenesis. The balance between osteogenesis and adipogenesis of bone marrow mesenchymal stem cells (BMSCs) plays a major role in the process of bone formation. Recently, intercellular communication between bone cells and surrounding cells has been gradually recognized, and macrophages on the surface of bone have been proven to regulate bone metabolism. However, the underlying mechanisms have not been fully elucidated. Recent studies have indicated that exosomes are vital messengers for cell-cell communication in various biological processes. In this experiment, we found that exosomes derived from M2 macrophages (M2D-Exos) could inhibit adipogenesis and promote osteogenesis of BMSCs. M2D-Exo intervention increased the expression of miR-690, IRS-1, and TAZ in BMSCs. Additionally, miR-690 knockdown in M2 macrophages with a miR-690 inhibitor partially counteracted the effect of M2D-Exos on BMSC differentiation and the upregulation of IRS-1 and TAZ expression. Taken together, the results of our study indicate that exosomes isolated from M2 macrophages could facilitate osteogenesis and reduce adipogenesis through the miR-690/IRS-1/TAZ axis and might be a therapeutic tool for bone loss diseases.

## Introduction

The skeletal system undergoes constant remodeling through functional changes in osteocytes, osteoclasts, and osteoblasts throughout life (1). The orderly progress of bone reconstruction is a complex process that involves many factors, including enzymes, hormones, and cytokines (2). Once the balance of bone metabolism is disrupted, a variety of bone disorders will develop. Bone marrow mesenchymal stem cells (BMSCs) are stromal stem cells that are derived from bone marrow and possess multiplex differentiation potential (3). Osteoblasts mature from BMSCs to osteoprogenitor cells, to osteoblast precursors, and ultimately to osteoblasts. Therefore, BMSCs play a critical role in bone formation (4). In recent decades, the osteogenic and adipogenic properties of BMSCs have been found to maintain a dynamic balance (5–8). Thus, regulating the balance between osteogenesis and adipogenesis may be a potential treatment strategy for bone loss diseases.

Recently, our understanding of the bone system has moved beyond bone cells. The crosstalk between bone cells and surrounding cells as well as the relationship between the bone and bone microenvironment has been gradually recognized and regarded as a relatively important factor in bone metabolism (9). Among these factors, the effect of macrophages on bone has attracted increased attention. Osteal macrophages are located on the bone surface and are tightly related to the osteogenesis of BMSCs (10). As early as 2002, Champaign found that macrophages could promote the osteogenic differentiation of BMSCs (11). A recent study suggested that human bone marrow mesenchymal stem cells showed stronger proliferation and osteogenic differentiation when cocultured with inactive human monocytes (12). Polarized macrophages can be divided into the M1 type and M2 type. M1 macrophages, positive expression of CD86, can produce tumor necrosis factor-α (TNF-α), induced nitrogen monoxide synthase (iNOS), Interleukin-12 (IL-12), and other proinflammatory cytokines to induce immune response. M2 macrophages, positive expression of CD206, contribute to tissue repair and anti-inflammatory functions. Recently, M2 macrophages have been gradually recognized as a positive regulator of bone formation during fracture healing (13). However, the underlying mechanism of this beneficial effect remains elusive. In addition, whether M2 macrophages can regulate the balance of osteogenic and adipogenic differentiation of BMSCs needs to be further explored.

Exosomes are extracellular vesicles formed by invagination or endocytosis that contain various types of biological information (14). Recent studies have found that exosomes play a vital role in bone diseases (15, 16). In a rat calvarial defect model, macrophage exosomes could mediate bone regeneration (17). However, it has also been reported that exosomes from M0, M1, and M2 macrophages might have different effects on the osteogenic differentiation of BMSCs (18, 19). This may be due to the different information substances contained in different exosomes. A recent study has shown that M2 exosomes are rich in miR-690 and could regulate insulin sensitivity (20). Insulin receptor substrate 1 (IRS-1) is the major signaling adapters in insulin/IGF-1 pathways and has been proved to play an important role in bone metabolism (21, 22). Our previous study found that IRS-1 regulates the osteogenic and adipogenic differentiation of BMSCs through Transcriptional co-activator with PDZ-binding motif (TAZ) (23, 24). TAZ could be combined with peroxisome proliferator-activated receptor γ (PPARγ) and Runt-related transcription factor 2 (Runx2) to regulate the balance between osteogenesis and adipogenesis (25). In this study, we explored whether exosomes derived from M2 macrophages could regulate the balance between osteogenesis and adipogenesis through miR-690-IRS-1-TAZ.

## Materials and Methods

### Cell Culture

All the experiments in the present research were approved by the Third Hospital of Hebei Medical University. The macrophage cell line RAW264.7 was incubated in α-MEM culture medium containing 10% fetal cattle serum (FBS) and 1% penicillin-streptomycin. For induction of M2 macrophage differentiation, RAW264.7 cells were cultured with 20 ng/ml Interleukin-4 (IL-4) for 24 h. Ten BALB/c male mice were obtained from the animal experimental center of Hebei Medical University and were used to separate BMSCs. Briefly, primary BMSCs were isolated from bone marrow and subsequently cultured in DMEM complete medium. For induction of osteogenesis, BMSCs were grown in osteogenic medium containing 10 mM β-glycerophosphoric acid, 10 nmol/L dexamethasone, and 50 μg/ml ascorbic acid. Adipogenesis was induced by 10 mg/ml insulin, 500 mmol/L methyl-isobutylxanthine, and 1 μmol/L dexamethasone. All cells were cultured at 37°C with 5% CO_2_.

### Identification of M2 Polarization Macrophages and Exosome

After RAW264.7 cells were cultured with 20 ng/ml Interleukin-4 (IL-4) for 24 h, the positive surface markers CD206 (BioLegend, San Diego, CA, USA) was analyzed by flow cytometry analysis.

After M2 differentiation of macrophages was induced, the MinuteTM efficient exosome precipitation reagent purchased from Inent Biotechnologies Company was used to obtain the exosomes according to the instructions. In short, the culture medium of M2 macrophages was harvested after culture in serum-free medium for 15 h. Then, cells were removed from the samples by low-speed centrifugation (5 min, 1,000 g), and the culture medium was obtained for further separation. The collected media was subsequently incubated with exosome precipitant overnight at 4°C. Next, the samples were centrifuged for 1 h at 10,000 g, and the supernatant was removed. The isolated exosomes were harvested and stored at −80°C for future use.

For further identification, the morphology and diameter of exosomes were characterized by transmission electron microscopy (Hitachi HT7700 TEM, Tokyo, Japan). Twenty microliter exosome samples were aspirated with a pipette gun and placed in carbon membrane copper mesh for 5 min. Then, the excess liquid was aspirated with filter paper. The sample was subsequently stained with 2% phosphotungstic acid for 2 min. The excess liquid was absorbed with filter paper. Finally, images of exosomes were collected by TEM. The number and size of exosomes were characterized by nanoparticle tracking analysis (NTA) using NanoSight NS-300. In addition, CD63 and CD81, exosomal specific surface proteins, were detected by western blotting.

### Exosome Uptake Assay

According to the instructions, exosomes were labeled with PKH26 (Sigma-Aldrich, Germany). First, 30 μl of exosomes was diluted in 1 ml of diluent C and 6 μl of PKH26 dye for 5 min. Then, 10% bovine serum albumin (BSA) was added to neutralize the excess dye, followed by washing in PBS for 60 min. Finally, BMSCs were treated with the labeled exosomes and analyzed by a fluorescence microscope.

### miRNA Inhibitor and Si-IRS-1 Plasmids Transfection

Cells were transfected with 30 nM miR-690 inhibitor or 30 nM inhibitor negative control (inhibitor NC) using Lipofectamine 3000 (Thermo Fisher Scientific, USA) based on the manufacturer’s protocol. Briefly, miR-690 inhibitor or inhibitor NC was mixed with Lipofectamine 3000 for 20 min separately before being cultured with cells. Cells were collected for further miRNA analysis at 48 h post-transfection. The miR-690 inhibitor and inhibitor negative control were all obtained from Zhongshi Tongtru (Tianjin, China). The sequences were as follows: miR-690 inhibitor 5’-UUUGGUUGUGAGCCUAGCCUUU-3’ and miR-690 inhibitor NC 5’-CAGUACUUUUGUGUAGUACAA-3’.

BMSCs were transfected with Si-IRS-1 or control plasmid (Genechem, Shanghai, China) using Lipofectamine 3000 after reaching 80% confluence, in accordance with manufacturer’s instructions.

### Alizarin Red Staining (A-R Staining)

A-R staining was performed after 2 weeks of osteoblast induction. In short, BMSCs were fixed with 4% paraformaldehyde (Solarbio, China) for 10 min after being washed twice with PBS. Then, 1% A-R staining solution was incubated for half an hour. Finally, red mineralized nodules were observed. In addition, the absorbance at 570 nm was subsequently measured *via* a microplate spectrophotometer (BioTek Instruments, San Jose, CA, USA).

### Oil Red O staining

Lipid droplet formation was detected by oil red O staining after culture in adipogenic medium for 2 weeks. Briefly, BMSCs were washed twice with PBS followed by fixation with 4% paraformaldehyde for 10 min. Then, oil red O staining solution was added to stain the cells for half an hour. Finally, the formation of lipid droplets was captured by a microscope. Moreover, the absorbance at 520 nm was subsequently analyzed.

### Real-Time Reverse Transcription Polymerase Chain Reaction (Real‐Time RT‐PCR)

For the analysis of mRNA, total RNA was extracted using TRIzol^®^ reagent (Tiangen, Beijing, China) before reverse transcription into cDNA *via* a RevertAid™ First Strand cDNA synthesis kit (Thermo, Waltham, USA) based on the instructions. Then, RT-PCR analysis was performed according to the protocols for Tiangen SuperReal PreMix Plus.

For the analysis of miRNA, microRNA assay kits were purchased from Zhongshi Tongtru (Tianjin, China) and used following the manufacturer’s instructions.

**Table 1** shows the PCR primer sequence, which is designed by primer software.

**Table 1:** Primers used for RT-PCR.

Gene	Forward Sequence (5′—>3′)	Reverse Sequence (5′—>3′)
GAPDH	AGTTCAACGGCACAGTCAAGG	AGCACCAGCATCACCCCAT
IRS-1	CCTGACATTGGAGGTGGGTC	TTACCACCACCGCTCTCAAC
TAZ	GTCACCAACAGTAGCTCAGATC	AGTGATTACAGCCAGGTTAGA AAG
RUNX2	GGACTGGGTATGGTTTGTAT	GCTGAAGAGGCTG TTTGA
OCN	ACCACATCGGCTTTCAGG	CATAGGGCTGGGAG GTCA
C/EBPβ	GCGGGGTTGTTGATGTTT	CTTTAATGCTC GAAACGG
PPARγ	CCTTGCTGTGGGGATGTCTCA	CTCCTTCTCGGCCTGTGGCAT
miR-690	5’-CTCAACTGGTGTCGTGGAGTCGGCAA TTCAGTTGAGTTTGGTT- 3’	
U6	5’-AACGCTTCACGAATTTGCGT-3′	

### Western Blotting

RIPA lysis buffer containing 1% protease inhibitor (Zhongshi Tongtru, Tianjin, China) was used to obtain total protein from the samples. Then, 20 μg protein was separated *via* SDS-PAGE and transferred to PVDF membranes before being blocked with 5% milk for 1 h. The PVDF membranes were stained at 4°C overnight with antibodies specific for IRS-1 (1:1,000, #2382, Cell Signaling, Beverly MA,USA), TAZ (1:1,000, #72804, Cell Signaling, Beverly MA,USA), OCN (1:1,000, ab274873, Abcam, Cambridge, England), RUNX2 (1:1,000, ab236639, Abcam, Cambridge, England), CEBPβ (1:1,000, ab32358, Abcam, Cambridge, England), PPARγ (1:1,000, ab272718, Abcam, Cambridge, England), and GAPDH (1:1,000, ab8248, Abcam, Cambridge, England). Finally, blots were stained with fluorescence secondary antibodies and detected with the Odyssey Infrared Imaging System (Li‐COR Biosciences).

### Statistical Analysis

Experimental data were obtained from at least three replicates and are shown as the mean ± standard deviation. Student’s t-tests and one-way ANOVA with Tukey’s *post hoc* test were used to compare the data between different groups as appropriate. p < 0.05 was considered statistically significant.

## Results

### Characterization of M2D-Exos

**Figure 1:**
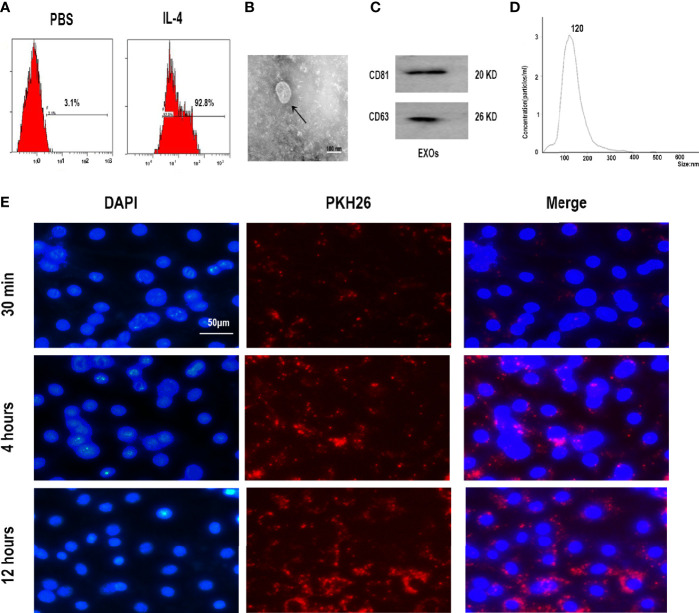
Characterization of M2 macrophages and exosomes derived from M2 macrophages. **(A)** Expression of M2 marker CD206 in polarized RAW 264.7 cells analyzed by flow cytometry. **(B)** Morphology identified by TEM, scale bar:100 nm. **(C)** The surface biomarkers CD63, CD81 were analyzed by Western blotting assay. **(D)** Size distribution profiles of M2D-Exos was detected by nanoparticle tracking analysis. **(E)** The exosomes derived from M2 macrophages was marked with red fluorescence dye PKH26 and co-cultured with BMSCs, scar bar = 50 µm.

Compared to PBS-treated macrophages, IL-4-treated macrophages showed significant upregulation of CD206 (**Figure 1A**), suggesting that the RAW264.7 cells were successfully induced to M2 polarization after stimulation with IL-4.

Exosomes were first harvested from the supernatants of M2 macrophages *via* exosome separation reagent. TEM, NTA, and western blotting analyses were performed to identify the collected M2D exosomes. Typical round or cup-shaped exosomal structures were revealed by TEM (**Figure 1B**). Western blotting analysis further provided evidence that the isolated particles were positive for exosomal surface markers, including CD63 and CD81 (**Figure 1C**). NTA analysis revealed that the diameters of these exosomes ranged from 50 to 150 nm (**Figure 1D**).Therefore, these above analyses confirmed the successful collection of M2D-Exos.

Subsequently, to verify whether the M2D-Exos could be endocytosed by BMSCs, we labeled the M2D-Exos with PKH26 and further cocultured them with BMSCs. As shown in **Figure 1E**, PKH26-labeled exosomes were localized in the BMSC region, which exhibited efficient internalization of the M2D-Exos by BMSCs.

### M2D-Exos Facilitated BMSCs Osteogenesis *In Vitro*

**Figure 2:**
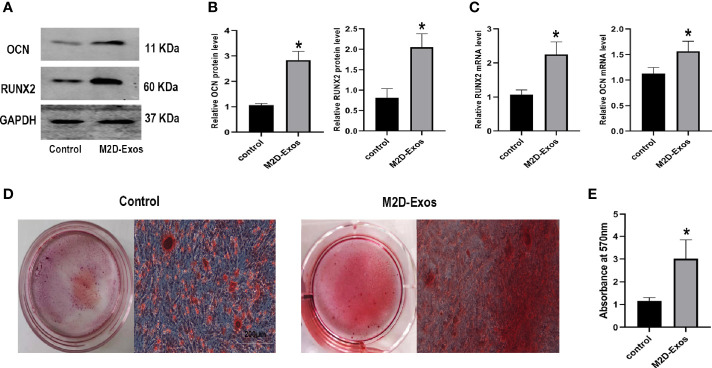
Exosomes derived from M2 macrophages enhanced osteogenic differentiation of BMSCs. **(A, B)** Osteogenic proteins, OCN and RUNX2, were measured by western blotting analysis and quantified, Control group was BMSCs treated in osteogenic inducer. **(C)** Osteogenic genes, OCN and RUNX2, were measured by RT-PCR analysis, Control group was BMSCs treated in osteogenic inducer. **(D)** Alizarin red staining was used to measure the formation of bone nodules in BMSCs after treated by PBS (control group) or M2D-Exos for 14 days. Scale bar = 200 µm. **(E)** The statistical data of Alizarin red-mediated calcium staining. *p < 0.05 compared to the control group. Control group was BMSCs treated in osteogenic inducer.

To further analyze the effect of M2D-Exo function on the osteogenesis of BMSCs, we performed western blotting, RT-PCR, and Alizarin red staining. The expression of the osteogenic differentiation-related proteins RUNX2 and osteocalcin (OCN) was upregulated after culture with M2D-Exos (**Figures 2A, B**). Consistent with this finding, significantly higher mRNA levels of RUNX2 and OCN were detected in the M2D-Exo group than in the control group (treated with PBS) (**Figure 2C**). In addition, the Alizarin red staining results revealed that the mineral deposition staining in the M2D-Exo-treated BMSCs was significantly larger than that in the control group (**Figures 2D, E**). The above findings indicate that M2D-Exos could increase the osteogenic differentiation of BMSCs.

### M2D-Exos Inhibited BMSCs Adipogenesis *In Vitro*

**Figure 3:**
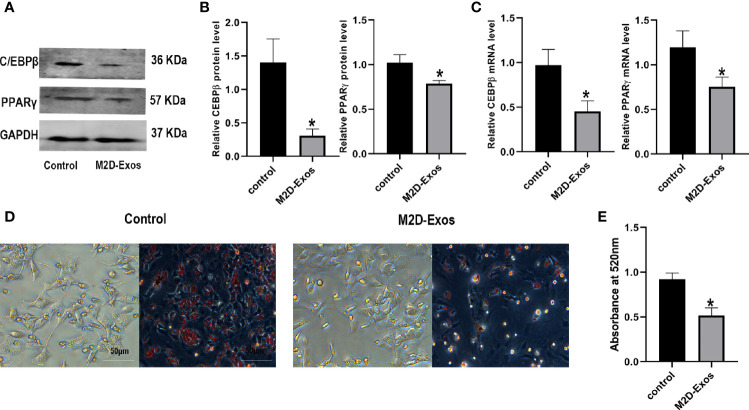
Exosomes derived from M2 macrophages inhibited adipogenic differentiation of BMSCs. **(A, B)** Adipogenic proteins, C/EBPβ and PPARγ, were measured by western blotting analysis and quantified. Control group was BMSCs treated in adipogenic inducer. **(C)** Osteogenic genes, C/EBPβ and PPARγ, were measured by RT-PCR analysis. Control group was BMSCs treated in adipogenic inducer. **(D)** Oil red O staining was used to measure the formation of lipid droplets in BMSCs after treated by PBS (control group) or M2D-Exos for 14 days. Scale bar = 50 µm. **(E)** The statistical data of Oil red O staining. *p < 0.05 compared to the control group. Control group was BMSCs treated in adipogenic inducer.

For further elucidation of the function of M2D-Exos in the adipogenesis of BMSCs, adipogenic differentiation-related genes, and proteins as well as the formation of lipid droplets were analyzed. The expression of CCAAT/enhancer binding protein β (C/EBPβ) and PPARγ was downregulated after treatment with M2D-Exos (**Figures 3A, B**). Similarly, the RT-PCR results showed lower mRNA levels of C/EBPβ and PPARγ in the M2D-Exo group compare to PBS-treated BMSCs (**Figure 3C**). In addition, oil red O staining revealed reduced formation of lipid droplets in the M2D-Exo-treated cells (**Figures 3D, E**), indicating an inhibitory effect of M2D-Exos on adipogenesis.

### M2D-Exos Delivered miR-690 Into BMSCs and Increased the Expression of IRS-1 and TAZ

**Figure 4:**
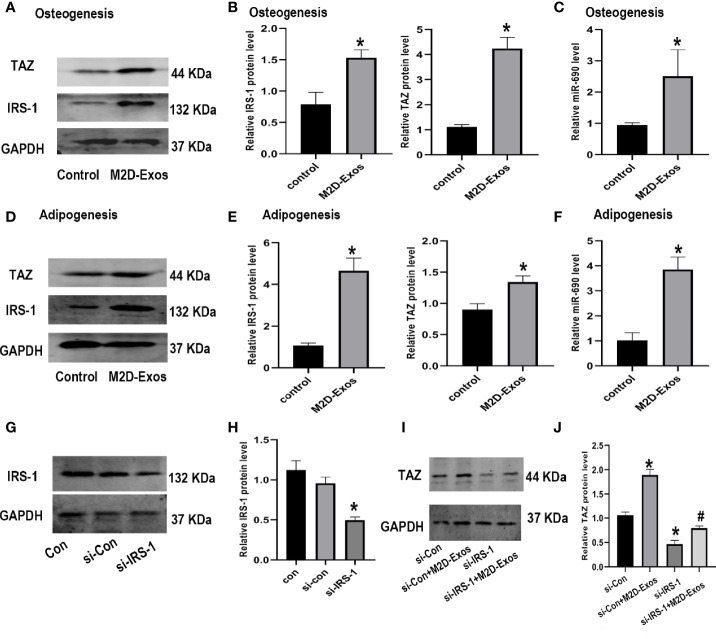
Exosomes derived from M2 macrophages increased the level of IRS-1 and TAZ protein of BMSCs. **(A, B)** IRS-1 and TAZ protein of BMSCs after treated with M2D-Exos or PBS during osteogenesis were measured by western blotting analysis and quantified. Control group was BMSCs treated in osteogenic inducer. **(C)** The level of miR-690 of BMSCs after treated with M2D-Exos during osteogenesis were measured by qRT-PCR analysis. Control group was BMSCs treated in osteogenic inducer. **(D, E)** IRS-1 and TAZ protein of BMSCs after treated with M2D-Exos or PBS during adipogenesis were measured by western blotting analysis and quantified. Control group was BMSCs treated in adipogenic inducer. **(F)** The level of miR-690 of BMSCs after treated with M2D-Exos during adipogenesis were measured by qRT-PCR analysis. Control group was BMSCs treated in adipogenic inducer. **(G, H)** IRS-1 expression was analyzed using western blotting after transfected with different plasmids. **(I, J)** TAZ expression was analyzed using western blotting after different treatments. *p < 0.05 compared to the control group.

To explore the mechanism involved in the regulation of the differentiation of BMSCs by M2D-Exos, we then detected the expression of miR-690, IRS-1, and TAZ. Western blotting analysis showed that TAZ and IRS-1 expression during osteogenesis was significantly increased in the M2D-Exo-induced BMSCs (**Figures 4A, B**). Moreover, M2D-Exo intervention led to an increased level of miR-690 during osteogenic differentiation (**Figure 4C**). During adipogenic differentiation, we also detected the expression of miR-690 and the IRS-1 and TAZ proteins. The results were consistent with those for osteogenic differentiation. M2D-Exo administration resulted in an increase in the levels of miR-690, IRS-1, and TAZ (**Figures 4D–F**). We then knocked down IRS-1 expression using si-IRS-1 plasmids (**Figures 4G, H**). Western blot analyses suggested that Si-IRS-1 significantly blocked the upregulation of TAZ expression after the intervention of M2D-Exos (**Figures 4I, J**). The above results indicated that M2D-Exos might up-regulate miR-690 of BMSCs and increase the expression of IRS-1 and TAZ to regulate the balance between adipogenesis and osteogenesis.

### miR-690 Inhibitor Abolished the Effect of M2D-Exos on Osteogenesis and Adipogenesis of BMSCs

**Figure 5:**
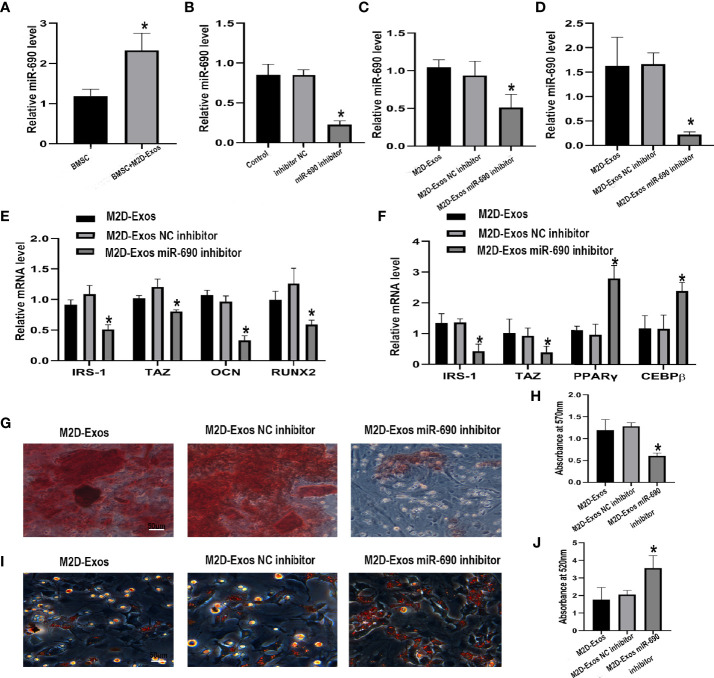
The inhibitor of miR-690 partially reversed the up-regulation osteogenesis and down-regulation adipogenesis induced by M2D-Exos. **(A)** The level of miR-690 in BMSCs after treated with M2D-Exos or PBS. *p < 0.05 compared to the BMSC group. **(B)** The level of miR-690 in M2 macrophages after treated with lipo3000, miR-690 inhibitor, or inhibitor control measured by qRT-PCR analysis. Control group was M2 macrophages treated with lipo3000. *p < 0.05 compared to the inhibitor control group. **(C)** The level of miR-690 in BMSCs after treated with M2D-Exos, miR-690 inhibited M2D-Exos, or inhibitor control M2D-Exos before cultured in osteogenic inducer were measured by qRT-PCR analysis. *p < 0.05 compared to the M2D-Exos NC inhibitor group. **(D)** The level of miR-690 in M2 macrophages after treated with lipo3000, miR-690 inhibitor, or inhibitor control before cultured in adipogenic inducer were measured by RT-PCR analysis. *p < 0.05 compared to the M2D-Exos NC inhibitor group. **(E)** IRS-1, TAZ, OCN, RUNX2 mRNA level of BMSCs after treated with M2D-Exos, miR-690 inhibited M2D-Exos, or inhibitor control M2D-Exos during osteogenesis were measured by qRT-PCR analysis. *p < 0.05 compared to the M2D-Exos NC inhibitor group. **(F)** IRS-1, TAZ, C/EBPβ, and PPARγ mRNA level of BMSCs after treated with M2D-Exos, miR-690 inhibited M2D-Exos, or inhibitor control M2D-Exos during adipogenesis were measured by qRT-PCR analysis. *p < 0.05 compared to the M2D-Exos NC inhibitor group. **(G)** A-R staining was used to measure the formation of bone nodules in BMSCs following treated by M2D-Exos, miR-690 inhibited M2D-Exos, or inhibitor control M2D-Exos for 14 days. Scale bar = 50 µm, **(H)** The statistical data of Alizarin red-mediated calcium staining. **(I)** Oil red O staining was used to measure the formation of lipid droplets in BMSCs following treated by M2D-Exos, miR-690 inhibited M2D-Exos, or inhibitor control M2D-Exos for 14 days. Scale bar = 50µm. **(J)** The statistical data of Oil red O staining. *p < 0.05 compared to the M2D-Exos NC inhibitor group.

To investigate whether miR-690 mediates the M2D-Exo-derived regulation of adipogenesis and osteogenesis, we first detected the expression of miR-690 in BMSCs after the intervene of M2D-Exos. The level of miR-690 was significantly increased after treated with M2D-Exos (**Figure 5A**), showing that M2D-Exos might transmit miR-690 to BMSCs. We then transfected a miR-690 inhibitor and inhibitor control into M2 macrophages. Compared with the inhibitor control, the miR-690 inhibitor significantly inhibited the level of miR-690 in M2D-Exos (**Figure 5B**). In addition, BMSCs were cocultured with miR-690 inhibitor-treated exosomes or miR-690 inhibitor control-treated exosomes, and the miR-690 inhibitor-treated exosome group showed a lower level of miR-690 than the inhibitor control group (**Figures 5C, D**). Furthermore, the miR-690 inhibitor attenuated the M2D-Exo-induced increases in IRS-1 and TAZ levels and the subsequent upregulation of osteogenic marker gene (RUNX2 and OCN) expression in osteogenic medium (**Figure 5E**). Similarly, the miR-690 inhibitor-treated exosomes inhibited the mRNA expression of IRS-1 and TAZ and increased the mRNA expression of C/EBPβ and PPARγ during adipogenic differentiation (**Figure 5F**). Furthermore, the results of Alizarin Red staining and oil red O staining were consistent with the real-time RT-PCR analyses and verified the mechanism by which M2D-Exos facilitated osteogenic differentiation and inhibited adipogenic differentiation by miR-690 (**Figures 5G–J**).

## Discussion

The close coupling of bone resorption and bone formation is vital to maintain bone remodeling. BMSCs have been confirmed to have an indispensable role in this continuous process (4). Recently, impaired bone formation with aging and diseases has been shown to be accompanied by decreased osteogenesis and increased adipogenesis (26, 27). Therefore, exploring the mechanism regulating the balance between osteogenesis and adipogenesis of BMSCs might be beneficial to further treatment for bone loss. Macrophages on the surface of bone may play an important role in the regulation of bone formation (18). In the present study, M2D-Exos promoted osteogenic differentiation and inhibited adipogenic differentiation of BMSCs through up-regulate miR-690, IRS-1, and TAZ.

The balance between osteogenesis and adipogenesis of BMSCs plays a vital role in bone remodeling (28). However, the differentiation of BMSCs is a complex process that is regulated by various factors. Recently, the bone microenvironment has been confirmed to have an indispensable function in osteogenesis (29). Macrophages are adjacent to bone. Macrophage-derived TNF-α can increase the chemotactic ability of osteoblasts (30). BMP-2 released by macrophages plays a vital role in the process of ossification through the Wnt and Wnt/LRP5 signaling cascades (31, 32). Macrophages can readily differentiate into different subtypes to regulate tissue homeostasis according to local cellular and secreted signals. In short, there are two polarized (M1 and M2) states and one unpolarized state (M0) of macrophages (32). M1 macrophages exert proinflammatory functions (33). In contrast, M2 macrophages contribute to the repair of tissue and exert anti-inflammatory functions (34). Different subtypes of macrophages exert different effects on bone formation. Recently, the positive role of M2 macrophages in bone remodeling during bone fracture has been gradually recognized. However, the exact mechanism is still unclear. Cell-conditioned medium generated by M2 macrophages influences the cellular behaviors of BMSCs (35), suggesting a paracrine effect of macrophages on BMSCs.

Exosomes have been demonstrated to be ideal transport devices for delivering regulatory substances to target cells. Cells receive messages within exosomes (including proteins and nucleic acids) through internalization to achieve communication between different types of cells. Recently, the regulatory effect of macrophage-derived exosomes on intestinal stem cells, endothelial cells, and tumors has been confirmed (36). Moreover, the role of M2D-Exos in bone metabolism has been gradually recognized (37). In the present study, PKH26 staining indicated that M2D-Exos can be internalized by BMSCs. M2D-Exo-treated BMSCs had a stronger osteogenic differentiation ability. This result is consistent with previous studies and suggests that exosomes are important mediators during M2 macrophage-induced osteogenesis of BMSCs (13). In addition, we further verified the effect of M2D-Exos on the adipogenic differentiation of BMSCs. The results revealed that M2D-Exos could inhibit adipogenic differentiation and the formation of lipid droplets in BMSCs. The above results suggest that M2D-Exos could regulate the balance of osteogenesis and adipogenesis, providing a potential therapeutic strategy for the treatment of bone loss diseases.

Small RNA molecules and miRNAs are involved in many diseases and processes, such as cancer and endocrine diseases (38). Evidence has also shown that exosomes can transport miRNAs to cells. A recent study showed that M2D-Exos are rich in miR-690 (20). In the present study, a higher level of miR-690 was also found in the M2D-Exo-treated BMSCs. Previous study has reported that miR-690 might up-regulate osteogenic differentiation by targeting NF-kappaB p65 (39). In addition, miR-690 could repress transcription factor CCAAT/enhancer-binding protein α (C/EBPα), which is an important regulation factor during adipogenesis (40). Therefore, miR-690 might mediate the regulation of M2D-Exos in the process of osteogenesis and adipogenesis. In the present study, we found that M2D-exos enhanced osteogenesis, decreased adipogenesis, and increased miR-690 level of BMSCs. At the same time, the levels of IRS-1 and TAZ were increased by M2D-exos intervention. A previous study showed that miR-690 can improve insulin sensitivity (20). As a major signaling adapter of the insulin/IGF-1 signaling pathway, IRS-1 stimulates a variety of downstream pathways to participate in the regulation of insulin resistance and cell differentiation (41). Our previous studies have shown that IRS-1 targets TAZ to facilitate osteogenesis and reduce adipogenesis in rat BMSCs (23, 24). TAZ is a transcription modulator that can influence stem cell fate determination through transcription factors (42). This molecule regulates signaling cascade genes to regulate the balance of osteogenesis and adipogenesis (43). In the present study, the level of TAZ and IRS-1 expression was increased after the administration of M2D-Exo. Knockdown of IRS-1 partially abolished the M2D-Exos induced elevation of TAZ in BMSCs. Thus, we hypothesized that M2D-Exos might regulate the differentiation of BMSCs *via* IRS-1/TAZ. To further verify the role of miR-690, we used a miR-690 inhibitor to reduce the level of miR-690 in M2D-Exos. The results showed that the expression of IRS-1 and TAZ was lower in the miR-690 inhibited-M2D-Exo-treated BMSCs than the inhibitor control-treated cells. Moreover, miR-690 inhibition in M2D-Exos partly counteracted the effect of M2D-Exos on promoting osteogenesis and inhibiting adipogenesis. Taken together, the results of our study indicate that exosomes isolated from M2 macrophages could facilitate osteogenesis and reduce adipogenesis through miR-690/IRS-1/TAZ.

The balance of osteogenesis and adipogenesis of BMSCs is vital for maintaining bone mass, which is regulated by various factors. Recently, macrophages had been found to play an important role in bone metabolism. However, the specific mechanism is still unclear. In the present study, we observed that exosomes derived from M2 macrophages could facilitate osteogenesis and reduce adipogenesis of BMSCs *in vitro*. This positive effect is at least partially mediated by miR-690, which is enriched in M2D-Exos and can upregulate the levels of IRS-1 and TAZ. Therefore, our findings suggested a potential role of M2D-Exos as a therapeutic tool for bone loss.

However, there are still some limitations in this study. First, we used RAW264.7 cells instead of primary macrophages. Second, we have not explored the direct target of miR-690 in BMSC. Third, this study is carried out *in vitro*. Next, we will further explore the direct target of miR-690 regulating IRS-1/TAZ in primary cells, and verify the positive role of M2D-exos in osteoporosis animal models.

## Data Availability Statement

The raw data supporting the conclusions of this article will be made available by the authors, without undue reservation.

## Ethics Statement

The animal study was reviewed and approved by Ethics Association of the Third Hospital of Hebei Medical University.

## Author Contributions

YL and ZL designed the study. ZL, SL, and YW collected the data, carried out the data analysis, and drafted the manuscript. All authors contributed to the article and approved the submitted version.

## Funding

Hebei Provincial Natural Science Foundation precision medicine joint fund cultivation project(H2020206314); Government funded clinical medicine talents training program in 2019.

## Conflict of Interest

The authors declare that the research was conducted in the absence of any commercial or financial relationships that could be construed as a potential conflict of interest.
